# Eat, breathe, sleep with Osteogenesis Imperfecta

**DOI:** 10.1186/s13023-021-02058-y

**Published:** 2021-10-18

**Authors:** Antonella LoMauro, Carlo Vittorio Landoni, Paolo Fraschini, Franco Molteni, Andrea Aliverti, Simona Bertoli, Ramona De Amicis

**Affiliations:** 1 Dipartimento di Elettronica, Informazione e Bioingegneria, Politecnico di Milano, Piazza Leonardo Da Vinci, 20133 Milano, Italy; 2 Valduce Hospital - Villa Beretta Rehabilitation Centre, Lecco, Italy; 3 IRCCS “Eugenio Medea” - Rehabilitation Unit, Bosisio Parini (LC), Italy; 4 International Center for the Assessment of Nutritional Status (ICANS), Department of Food, Environmental and Nutritional Sciences (DeFENS), University of Milan, Milan, Italy; 5 Obesity Unit and Laboratory of Nutrition and Obesity Research, Department of Endocrine and Metabolic Diseases, IRCCS Istituto Auxologico Italiano, Milan, Italy

**Keywords:** Osteogenesis Imperfecta, Spirometry, Opto-electronic plethysmography, Obstructive sleep apnea, Non-invasive ventilation, Fat mass, Body composition, Mediterranean diet, Scoliosis, AHI index

## Abstract

**Background:**

Although Osteogenesis Imperfecta (OI) affects the connective tissue causing extremely brittle bones with consequent skeletal deformities, it is important to go beyond bones. Indeed, the quality of life in OI does not only depend on bones status, as OI might affect also other important functions. We have therefore implemented a multidisciplinary study to assess lung function, breathing pattern, sleep quality and nutritional status in 27 adult OI type III and IV patients (median age: 34.6 years; 19 women; 14 type III).

**Results:**

According to nocturnal oxygen desaturation, two groups were identified: 13 patients with (OI_OSA, incidence: 48.2%) and 14 without (no_OSA) obstructive sleep apnea. The former was characterized by higher spinal and ribcage deformity, by more restrictive lung function, by paradoxical thoracic breathing in supine position, by rapid and shallow breathing, by higher body mass index, by longer neck and waist circumferences; by higher abdominal volume and by greater percentage of body fat mass, particularly localized in the trunk.

The best predictor of OI_OSA was the negative value of the supine ribcage contribution to tidal volume, followed by the ratio between the neck and the waist circumferences with body height and the supine thoraco-abdominal volumes phase shift angle.

**Conclusions:**

The pathophysiology of OI ensued a dangerous vicious circle, in which breathing, sleep and nutritional status are tightly linked, and they might all end up in negatively affecting the quality of life. The vicious circle is fed by some intrinsic characteristics of the disease (thoracic, cranial and mandibular deformities) and some bad daily habits of the patients (i.e. physical inactivity and low dietary quality). The former impacts on restricting the respiratory function, the latter makes Olers more prone to experience overweight or obesity. The main consequence is a high incidence of obstructive sleep apnea, which remains an underdiagnosed disorder in individuals with severe OI who are obese, with a neck to height ratio over than 31.6%, and characterized by paradoxical breathing in supine position. A multidisciplinary approach, including evaluations of breathing, sleep and nutrition, is required to better manage the disease and fulfil the maximizing well-being of OI patients.

## Background

Osteogenesis Imperfecta (OI) is a group of rare disorders occurring in 1 in 15,000 to 20,000 births [1]. It affects the connective tissue causing extremely fragile bones that break or fracture easily (brittle bones) and often without apparent cause [2]. There are four classical OI types according to severity based on clinical and radiological evaluation. Type III is the most severe form compatible with life with severely bone deformities and short height, while type I and IV are mild forms with moderate or less severe bone deformities [3].

The prevention, treatment and monitor of the respiratory function are important factors for prognosis in OI, as respiratory failure is the leading cause of death in these patients [4, 5]. For a respiratory point of view, OI is classified as a restrictive disease [6–9]. The two main factors predisposing to restricted respiratory problems in severe OI are severe kyphoscoliosis and structural modifications of the ribcage, with the most important effect being thoraco-abdominal asynchrony with thoracic paradoxical inward movement [8, 10, 11]. Because in the most severe OI form these alterations systematically occur at rest in supine position, we wonder if they may negatively affect sleep. Indeed, obstructive sleep apnea is recently shown to easily be left as an undetected disorder in OIers [12, 13]. This is a crucial problem, since sleep disordered breathing causes, among others, excessive daytime somnolence with negative effects on attention and vigilance, therefore reducing the quality of life.

In otherwise healthy patients, both respiratory function and sleep depend on nutritional status. Obesity induces restrictive respiratory pattern while promoting obstructive apneas during sleep [14–16]. Individuals with OI may experience overweight or even obesity secondary to physical inactivity, small body size and, possibly, inappropriate caloric intake and dietary quality [17, 18]. However, information on the nutritional status, body composition, and dietary habits of these patients is scarce, particularly in adult population. Collagen abnormalities, typical of OI, seem to imply also fat and fat free mass [19]. On the other hand, alterations in body composition is shown to be a strong risk factor for bone fractures in OI patients [18]. Particularly, truncal and abdominal fat mass is inversely related to bone mineral density [20] and sleep apnea because of the secretion of inflammatory cytokines causing increased bone resorption [21] and fat deposit in upper airway lumen and muscles [22]. Moreover, decreased mobility improves loss of lean mass, especially in terms of muscle mass, with increase of daily fatigue and exhaustion, therefore limiting daily activity, increasing obesity risk and ensuring a vicious circle [23]. In this scenario, a balanced diet rich in multifunctional nutrients (especially vegetables proteins, vitamins B, D and E, omega-3 fatty acids, oleic acid, selenium, calcium and polyphenolic compounds) with antioxidant and anti-inflammatory effects, as the Mediterranean pattern, could be recommended not only to improve body composition but also to potentiate pharmacologic treatment and physical activity [18].

Our main goal was to verify if the pathophysiology of OI in adult patients ensued a dangerous vicious circle, in which breathe, sleep and nutrition are tightly linked. We therefore implemented a multidisciplinary and multicentre study to assess lung function, breathing pattern, sleep quality and nutritional status on a group of OI patients.

## Methods

The study was conducted according to the statement of the Declaration of Helsinki and approved by the Ethical Board Committee of Valduce Hospital - Villa Beretta Rehabilitation Centre, Lecco, Italy.

### Patients

Patients were firstly enrolled among the members of As.It.O.I., the Italian Association of Osteogenesis Imperfecta. As.It.O.I., by posting an announcement of the association Facebook page.

Inclusion criteria were: confirmed diagnosis of OI type III and IV, stable condition, absence of severe cardio-respiratory pathologies, willingness to participate to the study and to travel to Milan for the tests. Informed consent was obtained from all study subjects or parents.

### Quality of life questionnaire

All patients filled in an Osteogenesis Imperfecta specific Quality of Life Questionnaire (OIQoL) [24, 25] comprising 33 questions grouped in six main themes (being safe and careful, reduced function, pain, fear, fatigue, independence). Each question was given a score ranging from 0 (worst scenario) to 4 (best scenario). For each theme, a global score was assigned as the mean of the corresponding questions.

### Spinal and ribcage deformity

Thoracic scoliosis was assessed through standard antero-posterior and lateral radiographic views of the entire spine and quantified using the Cobb method [26, 27].

The angle subtended at the sternal level on the transversal plane was computed to quantitatively describe the geometrical deformity of the ribcage, as previously described [8].

The spinal and ribcage deformity were combined by computing the mean value among the transversal and sagittal sternal angles and the Cobb angles of the thoracic and lumbar scoliosis.

### Nocturnal oxygen saturation

Median and nadir nocturnal oxygen saturation (SpO_2_) was measured using a digital pulse oximeter (Nonin, 8500 digital pulse oximeter Quitman, TX). The number of apnoea and hypopnea events per hour of sleep (AHI index) as well as the number of times per hour of sleep of oxygen desaturation (ODI index) were calculated. OSA was diagnosed as mild when 5 ≤ AHI < 15; moderate when 15 ≤ AHI < 30; severe when AHI ≥ 30 [28].

### Lung function and breathing pattern at rest

Measurement of forced vital capacity (FVC) and forced expiratory volume in 1 s (FEV_1_) were assessed through spirometry; while total lung capacity (TLC) through the nitrogen washout technique (Vmax series 22, SensorMedics, Yorba Linda, CA).

**Fig. 1:**
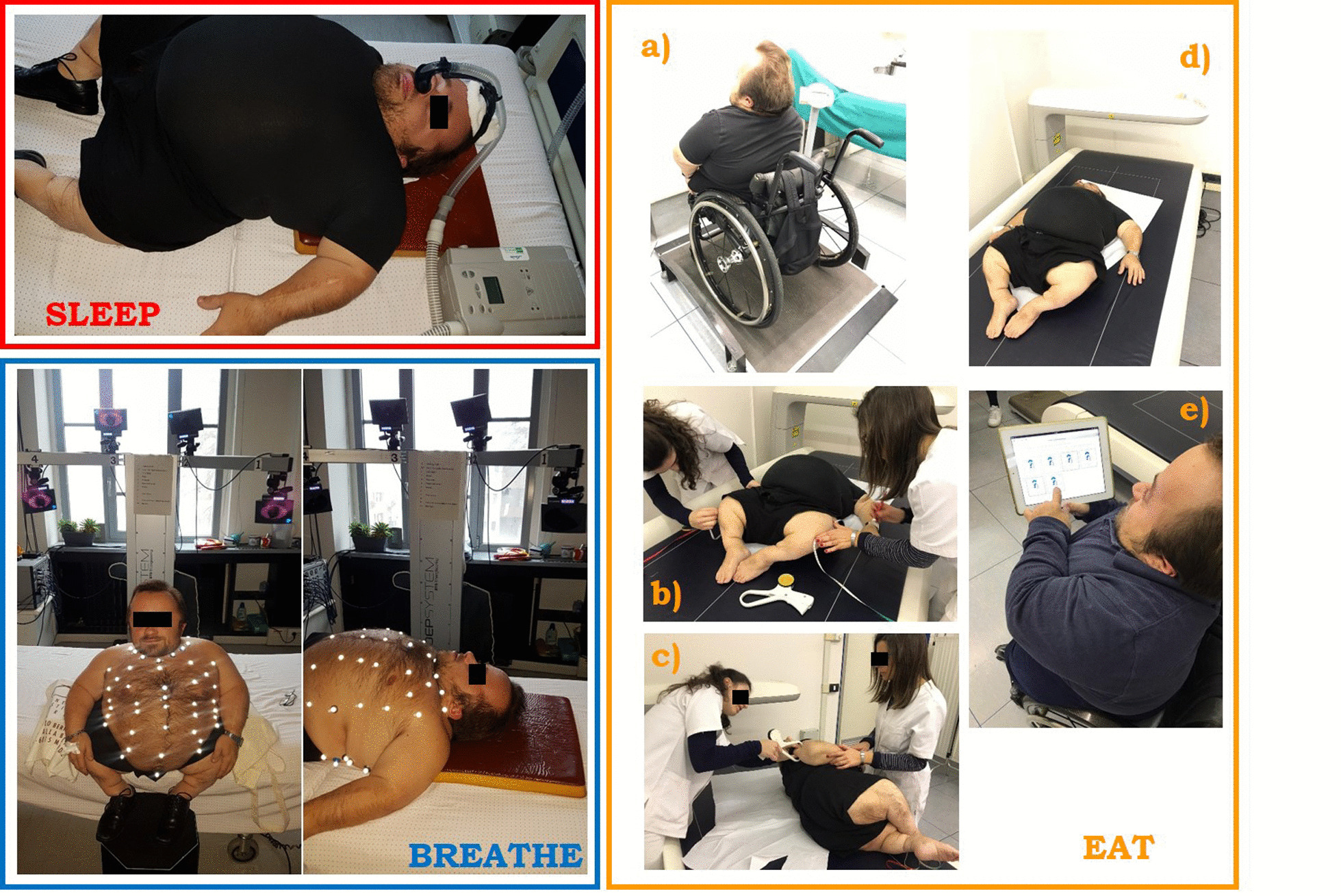
Protocol of measurement: sleep study in a patient already using nocturnal non-invasive mechanical ventilation (SLEEP, red); breathing pattern assessment through opto-electronic plethysmography (BREATHE, blue) and weight, height, anthropometric measurements, MED questionnaire (EAT, yellow)

Accurate assessment of ventilatory and thoraco-abdominal pattern during spontaneous quiet breathing was assessed by opto-electronic plethysmography (OEP System; BTS, Milan, Italy) in both seated and supine position (Fig. 1). Breathing frequency, tidal volume, minute ventilation (i.e. the product of the two), rapid and shallow breathing index (i.e. the ratio between breathing frequency and tidal volume), ribcage contribution to tidal volume (ΔV_RC_) and thoraco-abdominal phase shift angle were computed on an average breath of at least 45 s of spontaneous breathing. The abdominal volume at end expiration was also computed and expressed as percentage of total chest wall volume.

Because the output of OEP is the position of passive reflective markers put according to anatomical points, the trunk height was calculated as the distance between the marker at the sternoclavicular joint and the marker below the naval at the level of the iliac crest.

### Nutritional status

#### Anthropometric measurements

Anthropometric measurements were taken by the same operator, according to conventional criteria and measuring procedures [29]. Body weight (BW, kg) and body height (BH, cm) were measured to the nearest 0.1 kg and 0.5 cm, respectively. In subjects able to maintain the upright position, BW was measured on a beam balance scale and BH was measured with a stadiometer; in non-ambulant subjects unable to maintain the upright position, BW was measured using a digital wheelchair scale and BH was obtained measuring the supine length. BMI was calculated as BW (kg)/BH^2^ (m^2^).

Waist circumferences were measured midway between the lower rib margin and the superior anterior iliac spine using a horizontally applied non-stretch tape, and it was measured to the nearest 0.5 cm.

#### Dual-energy X-ray absorptiometry

DEXA scans (iDXA; General Electric, formerly Lunar Corp., Madison, WI) were used to obtain the body composition. DEXA provides measurements of soft tissue and bone for the total body and the sub regions (namely arm, trunk, leg) including fat mass (FM), lean body mass (LM) and bone mineral content (BMC). The fat mass percentage (FM%) was obtained as the ratio between total body fat mass and total body mass (fat + lean mass + bone mass of total body) multiplied for 100. The fat free mass (FFM) was calculated by adding BMC to LM. Fat mass (FMI) and lean mass indexes (LMI) were calculated by dividing FM and LM by the squared height, respectively. To investigate the proportion between LM and FM their ratio was also calculated.

The trunk region starts at the inferior edge of the chin and the lower borders intersect the middle of the femoral necks without touching the brim of the pelvis. It includes the neck, chest, abdominal and pelvic areas [30].

The scanning was done with patients lying supine on the table, their feet in a neutral position and arms resting along their sides, palms facing upwards. The DEXA scans, performed by well-trained and certified research staff, were all done using one device and the same software (enCORE, 2010), for an average measuring time of 10 min. The exposure to radiation was < 7 mSv. Daily quality-assurance was tested according to manufacturer directions. The DEXA scans were analysed using a custom-made software that allows body composition measurements in close relation to metal orthopaedic implants, by exclusion of no-osseous pixels.

#### Adherence to the Mediterranean diet and eating behaviour

Adherence to the traditional Mediterranean diet was assessed using a validated 14-item questionnaire [31]. The guidelines set out by the Prevención con Dieta Mediterránea (PREDIMED) study group (www.predimed.es) with some adaptation already employed in previous studies were used to obtain the Mediterranean score (MED score) [32, 33]. One point was attributed for each of the following: (1) olive oil as the main cooking fat; (2) olive oil ≥ 4 tablespoons/day; (3) vegetables > 2 servings/day (or ≥ 1 portion raw or salad) [31]; (4) fruit ≥ 3 servings/day; (5) red or processed meat < 1 serving/day; (6) butter or cream or margarine < 1/day; (7) sugar-sweetened beverages < 1/day; (8) wine ≥ 3 glasses/week; (9) legumes ≥ 3 servings/week; (10) fish/seafood ≥ 3 servings/week; (11) commercial sweets and confectionery < 3/week; (12) nuts ≥ 1/week; (13) white more than red meats (yes); and (14) use of sofrito ≥ 2/week. Participants with a MED score ≥ 9 points were considered as complying with a dietary pattern in accordance with the Mediterranean diet [31].

Eating behaviour was tested through the Binge Eating Scale (BES), a 16-item self-report questionnaire designed to capture the behavioural, the cognitive and the emotional features of regular objective compulsive overeating [34].

### Statistical analysis

Mann–Whitney rank sum test was used to assess differences between the two groups, which resulted non-normal distributed according to Kolmogorov–Smirnov test, with OSA status as independent factor (SigmaStat version 11.0; Systat Software, San Jose, Calif., USA).

Significance was set as *p* < 0.05. Data in the results section of the text are reported as median value (25th–75th percentiles).

For all the parameters that resulted significantly different between the two groups of patients, the receiver operating characteristic (ROC) curve was computed to illustrate their diagnostic ability in our binary classifier system.

The area under the ROC curve (AUC) was also calculated. AUC is an evaluation metrics for checking any classification model’s performance. The higher AUC, the better the model is at distinguishing between patients with OSA and no-OSA. AUC is considered excellent for values between 0.9 and 1, good between 0.8 and 0.9, fair between 0.7 and 0.8, poor between 0.6 and 0.7 and failed between 0.5 and 0.6 [35, 36].

Finally, the threshold of each considered parameter, which corresponds to the optimal operating point of the ROC analysis, was also derived.

## Results

### Patients

Forty-five patients answered to the announcement willing to participate to the study, but 18 did not meet the inclusion criteria, with logistic and independent travelling to Milan being the principal impediment.

Twenty-seven OI patients (19 women; 14 type III [3, 37, 38]) were therefore recruited for this study [age: 34.6 (31.1–44.6) years; height: 1.24 (1.0–1.4) m; weight: 46.8 (34.6–61.5) kg; BMI: 30.9 (25.0–35.7) kg/m^2^; body fat mass: 39.3 (34.7–47.3) %, the 51.5 (45.1–61.2) % of which located in the trunk, fat mass/fat free mass: 0.69 (0.55–0.94)].

According to nocturnal oxygen desaturation, two groups were identified (Table 1): 13 patients with obstructive sleep apnoea (OI_OSA; 7 women; 10 type III; 9 wheelchair bound patients; 4 patients under nocturnal non-invasive mechanical ventilation for a former diagnosis of obstructive sleep apnoea) and 14 patients without obstructive sleep apnoea (no_OSA; 12 women; 4 type III; 11 ambulant patients).

**Table 1:** OI quality of life, thoracic deformity and nutritional status

	no_OSA			OI_OSA			*P* value
	Median	25th p	75th p	Median	25th p	75th p	
Age (years)	32.7	17.4	44.4	39.3	34.3	49.0	0.094
*OI quality of life*							
Safety (/4)	3.50	3.38	3.50	3.50	3.25	3.50	0.945
Fatigue (/4)	3.50	3.00	3.50	3.00	3.00	3.50	0.345
Reduced function (/4)	3.50	3.00	3.50	3.50	3.00	3.50	0.892
Pain (/4)	3.00	2.50	3.50	3.00	2.50	3.50	0.653
Fear (/4)	3.50	3.38	3.50	3.50	3.00	3.50	0.710
Independence (/4)	3.50	3.50	4.00	3.50	3.00	4.00	0.575
*Thoracic deformity*							
Transversal sternal angle (°)	164.8	159.5	173.4	162.9	155.0	171.4	0.250
Sagittal sternal angle (°)	165.8	163.9	172.4	169.0	159.0	176.8	0.547
Thoracic scoliosis (Cobb angle)	30.0	20.0	30.0	35.0	30.0	40.0	**0.055**
Lumbar scoliosis (Cobb angle)	30.0	20.0	32.5	35.0	30.0	40.0	**0.048**
*Nutritional status*							
Weight (kg)	46.1	32.9	58.0	47.6	34.6	65.0	0.789
Height (m)	1.36	1.13	1.42	1.16	1.01	1.28	0.132
Neck circumference (cm)	34.5	32.3	35.5	38.8	38.0	41.0	**0.010**
Waist circumference (cm)	75.3	71.1	86.7	100.0	82.8	110.8	**0.039**
Abdominal volume (%chest wall)	25.8	24.7	33.3	35.3	33.3	42.2	**0.013**
Fat mass/fat free mass	0.62	0.49	0.74	0.89	0.61	1.14	**0.056**
LMI (kg/m^2^)	15.0	14.2	16.4	16.9	15.5	17.8	0.064
FMI (kg/m^2^)	8.4	7.6	13.0	14.8	10.7	21.9	**0.030**
LMI_TR_ (kg/m^2^)	163.9	148.5	187.8	198.8	171.2	218.4	**0.036**
FMI_TR_ (kg/m^2^)	96.0	68.0	148.9	176.1	121.6	282.8	**0.033**
MED score	6.00	5.00	6.75	6.00	5.00	7.00	0.564

### Quality of life

According to the OI Quality of Life questionnaire, no differences were found between the two groups in terms of safety, reduced function, pain, fear, fatigue and independence (Table 1).

### Spinal and ribcage deformity

While sternal angles were similar between the two groups, both thoracic and lumbar Cobb angles were significantly higher in OI_OSA patients (Table 1). The mean values of sternal angles, thoracic and lumbar Cobb angles were significantly higher in OI_OSA (101, 100°–104°) compared to no_OSA (95, 92°–101°, *p* = 0.025) patients.

### Sleep

**Fig. 2:**
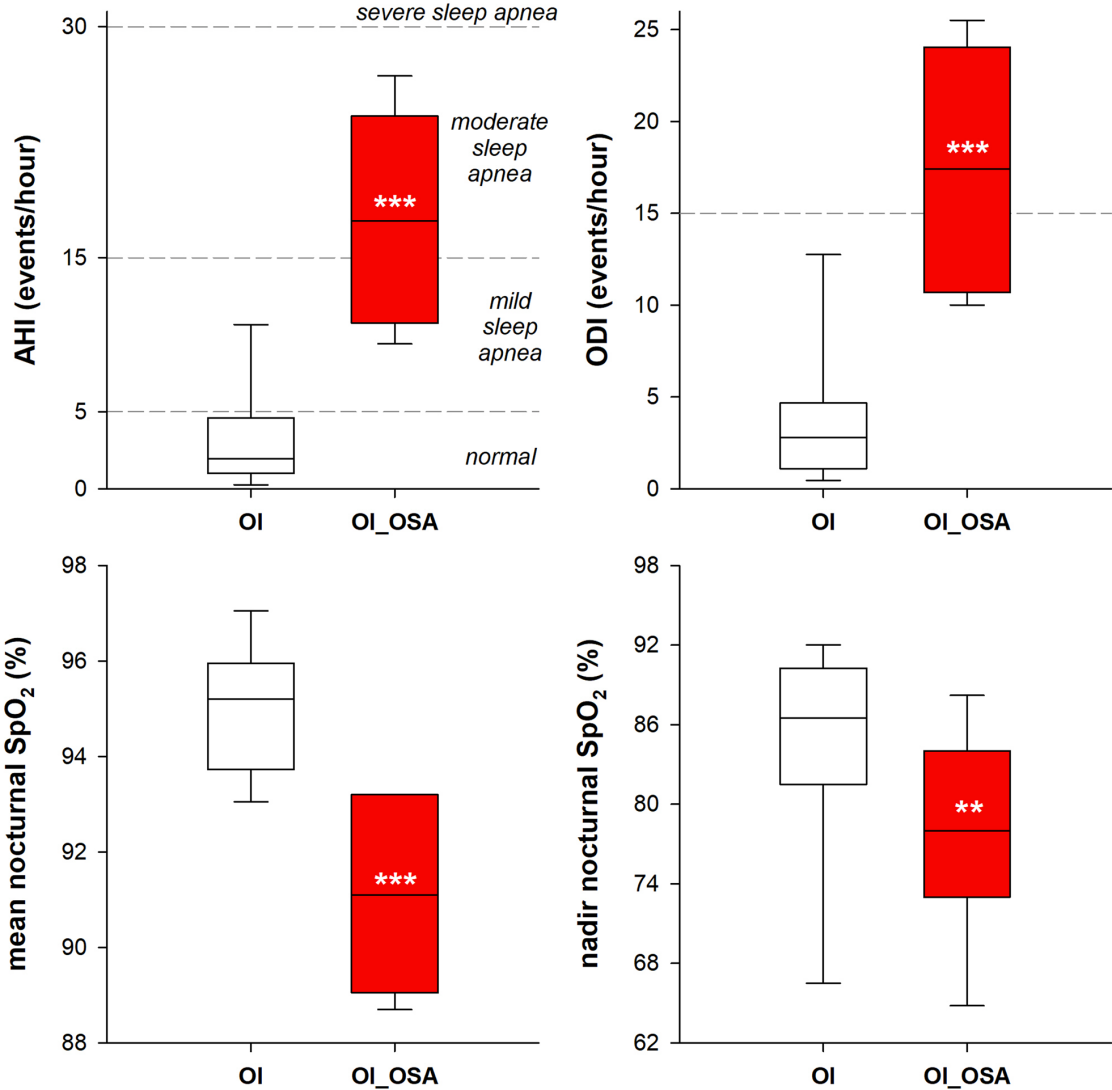
Box-plot representing the median (line within the box), the 10th (whisker below the box), 25th (boundary of the box closest to zero), the 75th (boundary of the box farthest from zero) and the 90th (whisker above the box) percentiles of AHI index (top left), ODI index (top right), mean (bottom left) and nadir (bottom right) nocturnal saturation in patients with (OI_OSA, red) and without (OI, white) obstructive sleep apnea. **p < 0.01; ***p < 0.001

By definition, OI_OSA patients were characterized by significantly higher AHI and oxygen desaturation index (ODI), and by significantly lower mean and nadir nocturnal oxygen saturation (Fig. 2).

### Breathe

**Fig. 3:**
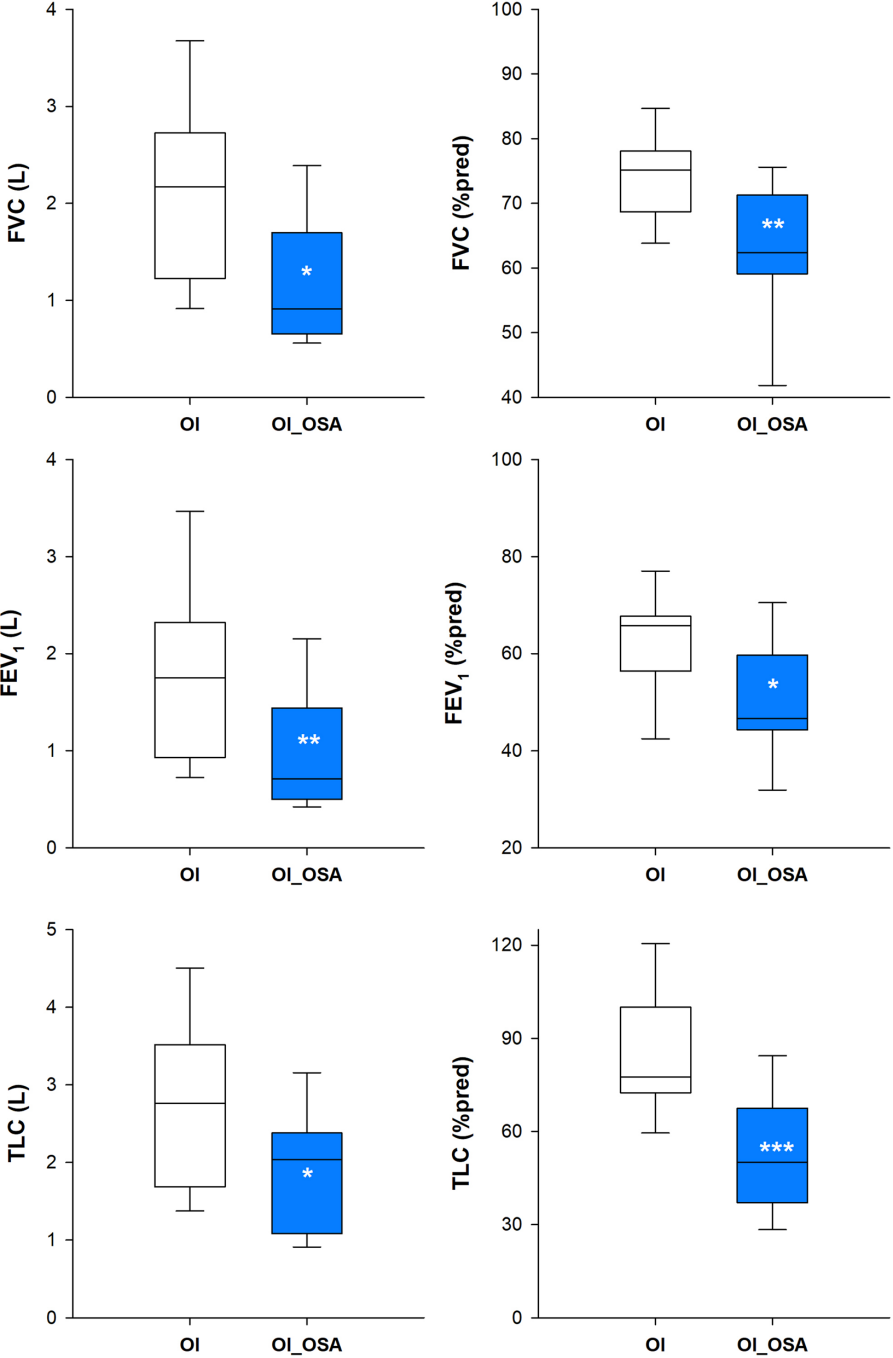
Box-plot representing the median (line within the box), the 10th (whisker below the box), 25th (boundary of the box closest to zero), the 75th (boundary of the box farthest from zero) and the 90th (whisker above the box) percentiles of forced vital capacity (top), forced expiratory volume in the first second (middle) and total lung capacity (bottom) in patients with (OI_OSA, blue) and without (OI, white) obstructive sleep apnea. Data are expressed both as litres (left panels) and as percentage of the predicted values (right panels). *p < 0.05; **p < 0.01; ***p < 0.001

**Fig. 4:**
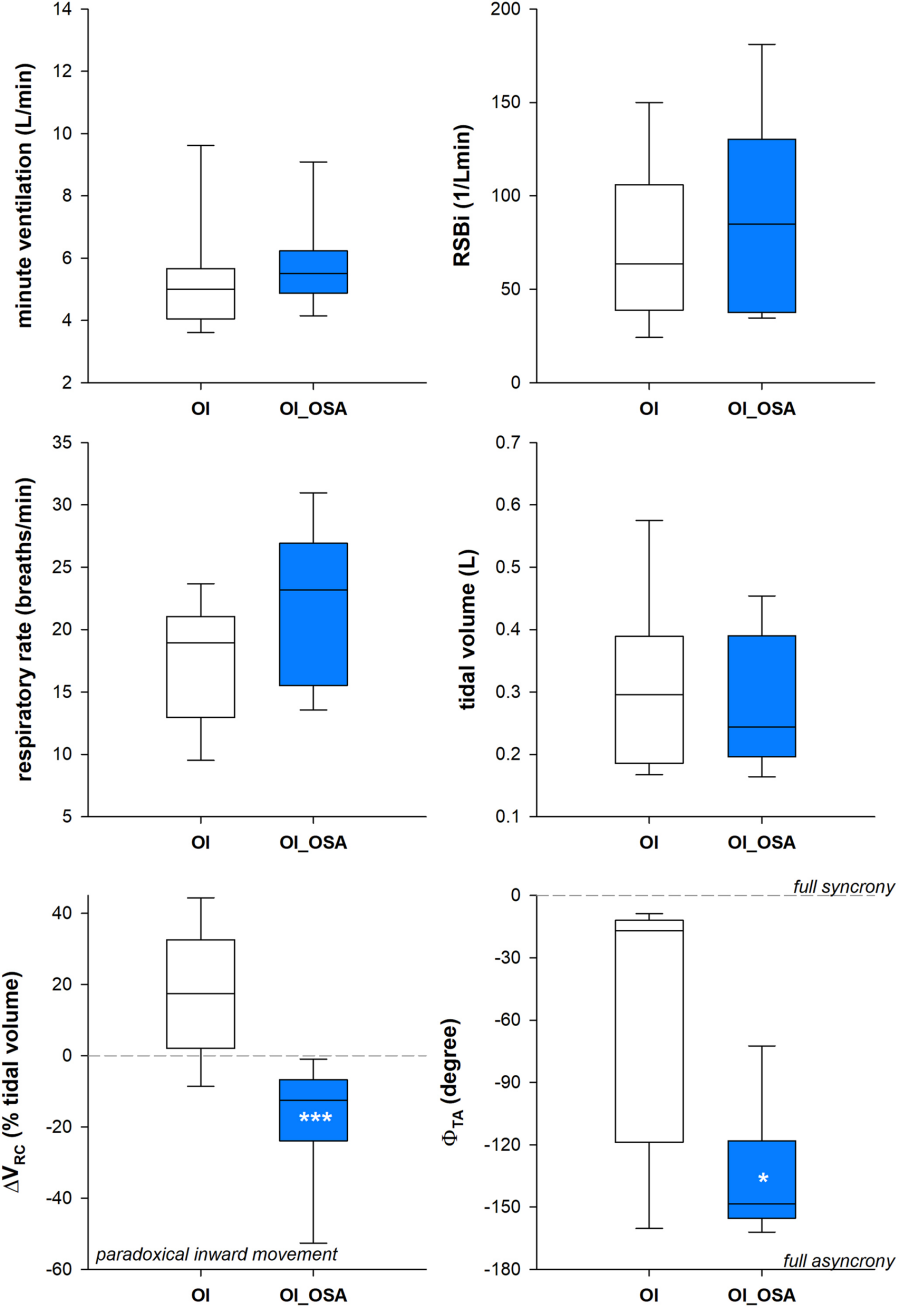
Box-plot representing the median (line within the box), the 10th (whisker below the box), 25th (boundary of the box closest to zero), the 75th (boundary of the box farthest from zero) and the 90th (whisker above the box) percentiles of minute ventilation (top left), rapid and shallow breathing index (top right), breathing frequency (middle left), tidal volume (middle right), pulmonary ribcage percentage contribution to tidal volume (bottom left) and thoraco-abdominal phase shift angle (bottom right) at rest in supine position in patients with (OI_OSA, blue) and without (OI, white) obstructive sleep apnea. *p < 0.05; ***p < 0.001

**Fig. 5:**
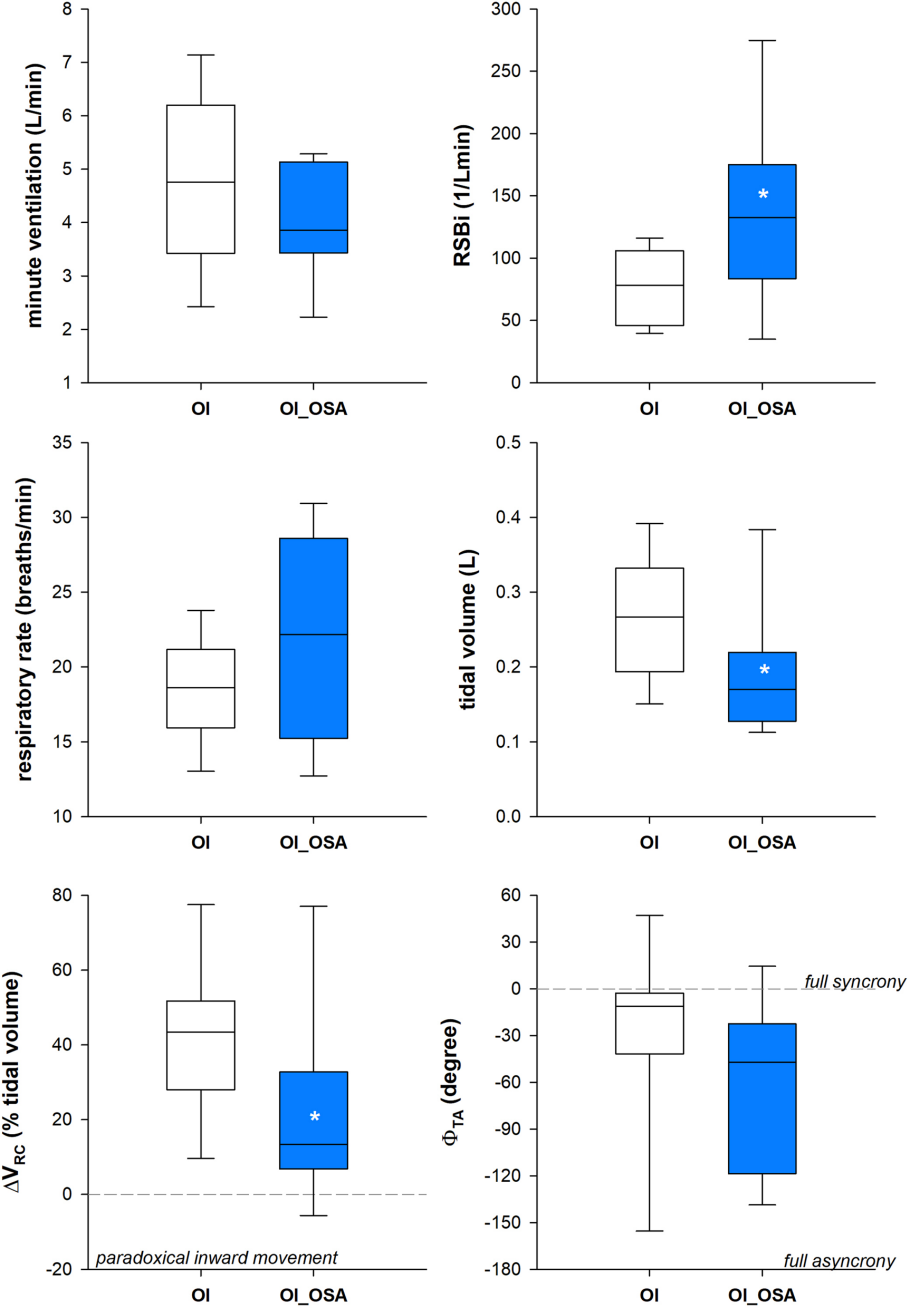
Box-plot representing the median (line within the box), the 10th (whisker below the box), 25th (boundary of the box closest to zero), the 75th (boundary of the box farthest from zero) and the 90th (whisker above the box) percentiles of minute ventilation (top left), rapid and shallow breathing index (top right), breathing frequency (middle left), tidal volume (middle right), pulmonary ribcage percentage contribution to tidal volume (bottom left) and thoraco-abdominal phase shift angle (bottom right) at rest in seated position in patients with (OI_OSA, blue) and without (OI, white) obstructive sleep apnea. *p < 0.05; **p < 0.01

OI_OSA patients were characterized by lower forced vital capacity, forced expired volume in one second and total lung capacity when expressed as absolute and percentage value (Fig. 3). Despite similar ventilatory pattern between the two groups in supine position, OI_OSA patients breathed with a systematic inward inspiratory paradoxical movement of the ribcage, leading to almost paradoxical thoraco-abdominal breathing with the phase shift angle approaching 180° (Fig. 4). In seated position, OI_OSA patients breathed with higher rapid and shallow breathing index, because of lower tidal volume, due to reduced ribcage contribution, with consequent higher thoraco-abdominal phase shift angle (Fig. 5).

### Eat

**Fig. 6:**
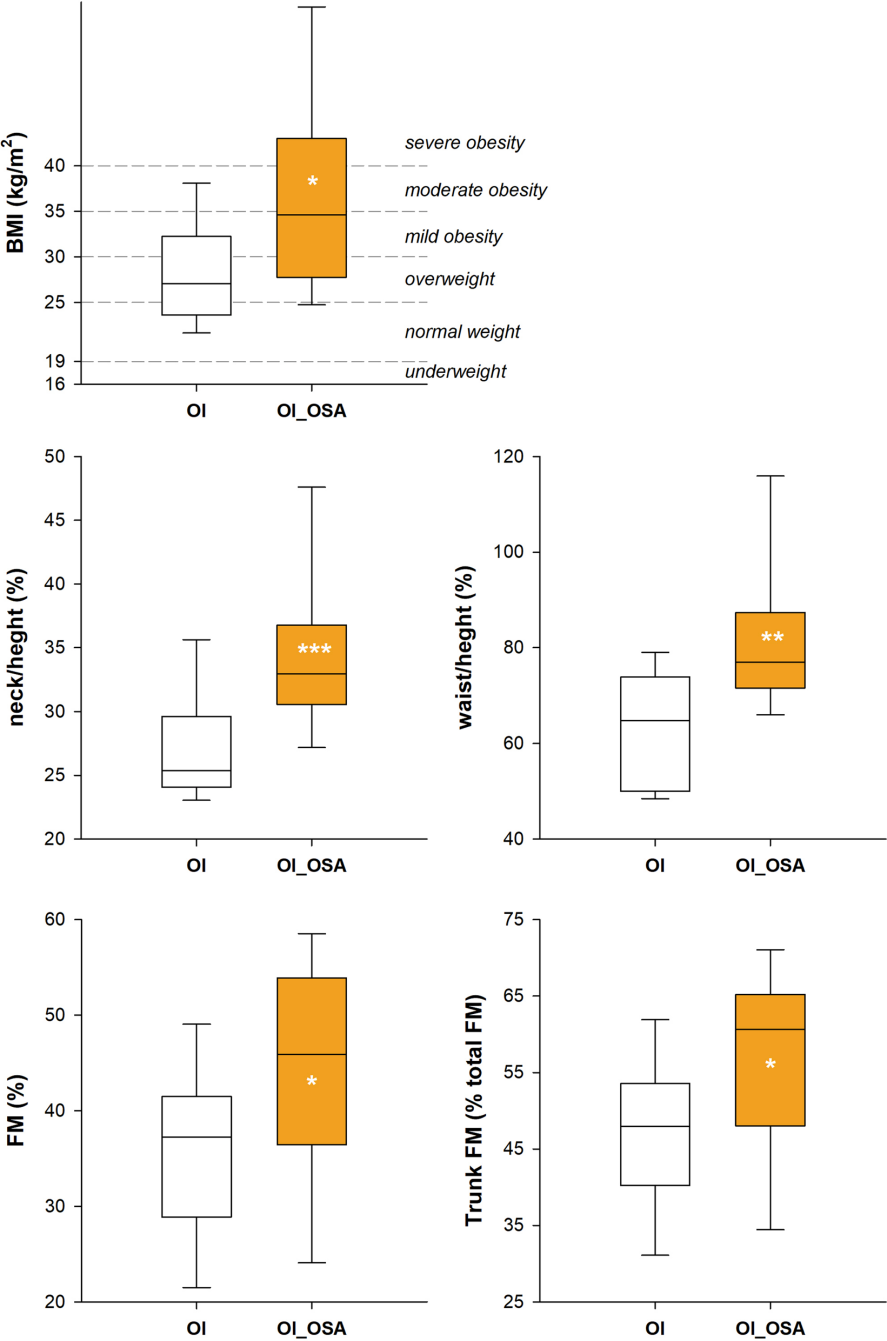
Box-plot representing the median (line within the box), the 10th (whisker below the box), 25th (boundary of the box closest to zero), the 75th (boundary of the box farthest from zero) and the 90th (whisker above the box) percentiles of body mass index (top left), neck circumference to height ratio (middle left), waist circumference to height ratio (middle right), total percentage of fat mass (bottom left) and the percentage of trunk fat mass (bottom right) in patients with (OI_OSA, yellow) and without (OI, white) obstructive sleep apnea. *p < 0.05; **p < 0.01; ***p < 0.001

Although weight and height were similar between the two groups (Table 1), body mass index was significantly higher in OI_OSA (60% of obesity in OI_OSA vs 46% in no_OSA; Fig. 6). In these patients, neck and waist circumferences were significantly longer when expressed both as absolute value (Table 1) and normalized according to body height (Fig. 6). Similarly, abdominal volume was higher in OI_OSA when expressed as percentage of total chest wall volume (Table 1). They also presented greater percentage of body fat mass, particularly localized in the trunk (Fig. 6), and, consequently, lower lean mass index.

Concerning dietary habits, 85% of OI_OSA showed a lower dietary quality (MED score < 9) compared to 71% of no_OSA. In general, 78% of all participants reported a low consumption of wholesome foods, such as fish, fruit, vegetables, legumes, nuts, and a higher tendency to the consumption of noxious foods, such as sugary drinks, red meat, and confectionery.

We did not report data on eating behaviour, since only three patients (11%) answered the BES questionnaire.

### Receiver operating characteristic (ROC) curve

Table 2 reports the area under the receiver operating characteristic (ROC) curve (AUC) and the threshold corresponding to the optimal operating point of the ROC analysis therefore relieving the best predictors of OSA in OI.

**Table 2:** Area under the curve (AUC) and threshold that corresponds to the optimal operating point (T) of the ROC analysis

	AUC	T
AHI (events/hours)	0.97	10.50
ODI (events/hour)	0.93	10.00
Mean SpO_2_ (%)	0.05	97.20
Nadir SpO_2_ (%)	0.13	93.00
FVC (L)	0.21	4.02
FVC (%)	0.15	85.17
FEV_1_(L)	0.19	3.56
FEV_1_ (%)	0.21	78.55
TLC (L)	0.24	4.72
TLC (%)	0.10	124.0
ΔV_RC_ supine (%)	0.93	-6.16
Φ_TA_ supine (°)	0.83	-126.5
RSBi seated (1/L min)	0.77	126.9
V_T_ seated (L)	0.28	0.44
ΔV_RC_ seated (%)	0.18	100.0
BMI (kg/m^2^)	0.75	34.60
Waist circumference (cm)	0.72	82.80
Neck circumference (cm)	0.79	38.20
Waist/height	0.84	70.69
Neck/height	0.88	31.64
FM (%)	0.69	43.00
Trunk FM (%)	0.74	54.83

A part form AHI and ODI, a negative value of the supine ribcage contribution to tidal volume resulted to be the best predictor of OSA in OI. The other best predictors were the ratio between the neck and the waist circumferences with body height and the supine thoraco-abdominal phase shift angle.

## Discussion

**Fig. 7:**
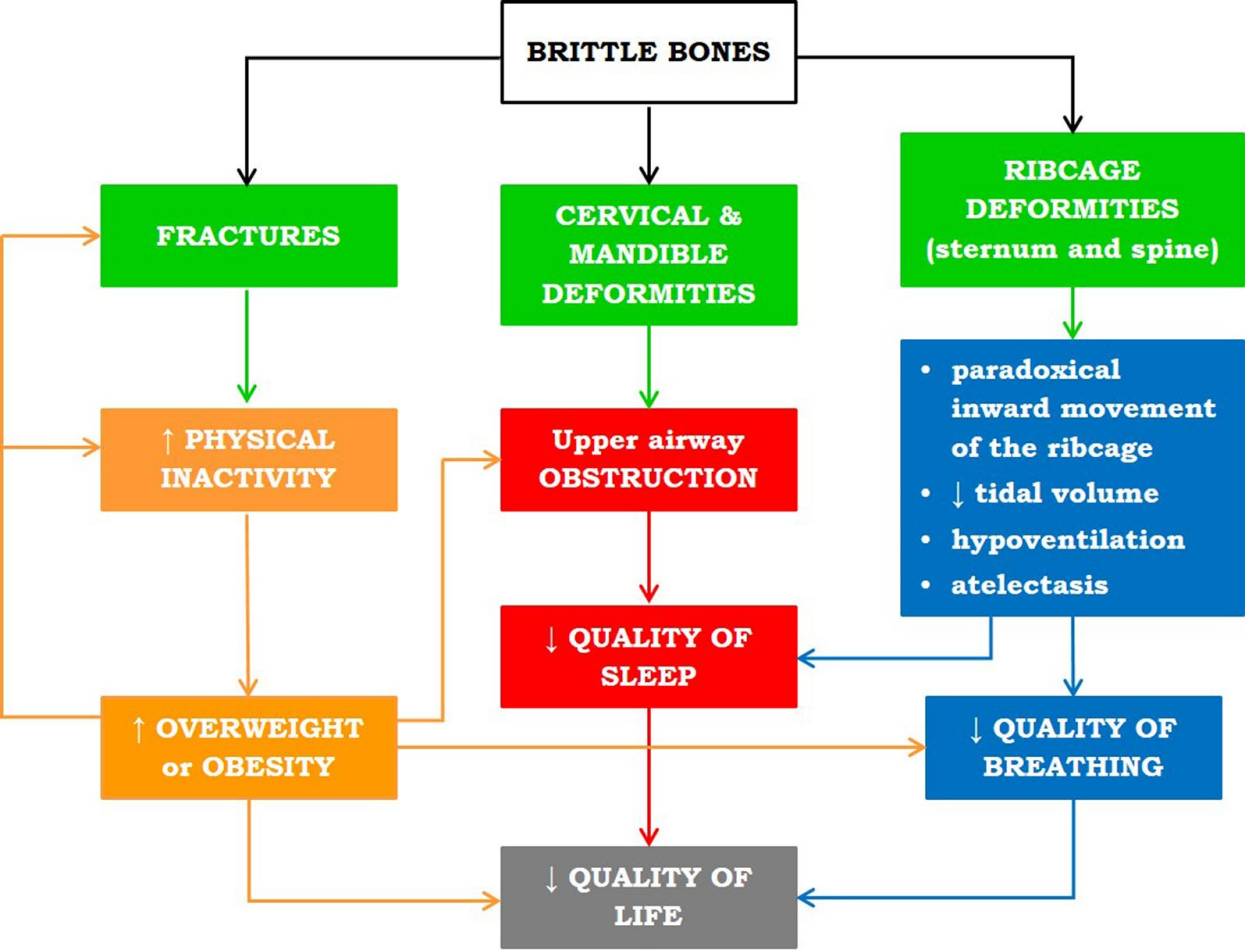
Diagram of the dangerous vicious circle, ensued by the pathophysiology of OI, in which breathing, sleep and body composition are tightly linked, and they all negatively affect the quality of life in OI patients.: ↓ reduced. ↑: increased

The pathophysiology of OI ensued a dangerous vicious circle, in which breathing, sleep and nutritional status are tightly linked, and they might all end up in negatively affecting the quality of life (Fig. 7). Obstructive sleep apnoea occurred in the 48.2% of our study population, the 70% of which was a new diagnosis because of the screening and not because of patients’ awareness of the problem. OSA was prevalent in subtype III obese patients who were characterized by more restrictive lung pattern, thoracic paradoxical movement in supine position and fat predominantly located around the neck and in the abdomen. Paradoxical breathing and neck circumference were the best discriminators for OI patients experiencing OSA.

OSA is a severe public health problem for the general population. It may increase the risks for hypertension, cardiovascular events, cerebrovascular accidents as well as for motor vehicle accidents and awakening headache [39]. OSA ultimately impacts on work performance [40] and depresses the quality of life. OSA risks tend to be underdiagnosed in OIers, although they frequently report fatigue and sleep disturbances [41]. Indeed, the incidence of OSA that we found in our population was very high, with the majority of OSA patients not being aware of the problem, as confirmed by the quality of life questionnaire. Our incidence was similar to that already found in another OI population [12], similar to the general obese population (~ 45%) and higher than the general population (~ 25%) [42].

However, OIers experiencing OSA are not “just” obese patients with OSA. OI patients are also characterized by a progressively severely reduced level of mobility of the thorax due to the fragility and the deformation not only of the bones, but also of the ligamentous and articular structures. For this reason, these patients deserve dedicated studies, while extrapolating the results from general and/or paediatric OI populations risks to underestimate the problem. In addition to the altered ribcage structure, OI patients might also experience altered gas exchange because of an intrinsic deficit of the collagen in the lung tissue [43]. The quality of sleep is strongly correlated with pulmonary function and breathing pattern and they could all depend on body weight. In addition, severe kyphoscoliosis and structural modifications of the ribcage (namely, *pectus carinatum*, horizontal and brittle ribs) are two typical OI characteristic that represent two main factors predisposing to respiratory problems, particularly in the most severe form. The former limits thoracic movements and lung expansion, the latter has important consequences on ribcage respiratory function. We believe that this interaction is mediated more by the combination of spinal and ribcage deformities rather than by the single one. Indeed, we have previously shown that the altered breathing pattern in severe OI worsens with age as a consequence of the combination between the congenital *pectus carinatum* and spinal deformity that starts in later childhood to develop thereafter [10]. The results of the present study seem to go towards this direction, with the combining index of deformity significantly being higher in OSA group. These patients were characterized by a more severe restricted spirometric pattern and by thoraco-abdominal asynchrony with paradoxical inward movement of the ribcage during spontaneous breathing at rest particularly in supine position.

Besides thoracic deformities, nutritional status played an additional role that worsened this scenario. Significantly higher BMI, body fat mass, trunk fat mass, neck and waist circumferences as well as abdominal volume characterized adult OIers suffering from sleep apnoea. These results are in line with what previously found in children with OI [44]. In that study, the authors conclude that symptoms suggesting obstructive sleep disorders should be searched especially in children with compromised autonomy, high BMI, trunk deformations, and severe OI type [44].

The mechanical effect of the excessive adipose tissue located around the neck and the abdomen makes: (1) lung capacities further reduce; (2) chest wall compliance further decrease; (3) work of breathing further increase; (4) oxygen cost of breathing increase; (5) central respiratory drive reduce; (6) expiratory flow limitation increase; (7) inspiratory muscle strength decrease and (8) upper airways obstruction increase [14–16].

Olers are more prone to experience overweight or even obesity secondary to physical inactivity [45], small body size and inappropriate dietary habits [18, 46, 47]. This happens not only in paediatric patients [19], but also in adulthood as shown in this study. Body composition is anecdotally shown to be a strong risk factor for bone fractures in OI patients. Fat negatively affects bone physiology, while poor muscle mass and strength correlate with incremental risk of fractures, but also with daily fatigue and exhaustion, limiting daily activity [18, 20, 21].

In order to act on this vicious circle of “eat, breathe and sleep in OI”, a multidisciplinary approach is required to better understand and manage the disease and fulfil the maximizing well-being of OI patients. This approach may include the use of nocturnal non-invasive mechanical ventilation (nNIV) and/or the onset of a controlled dietary regimen.

**Fig. 8:**
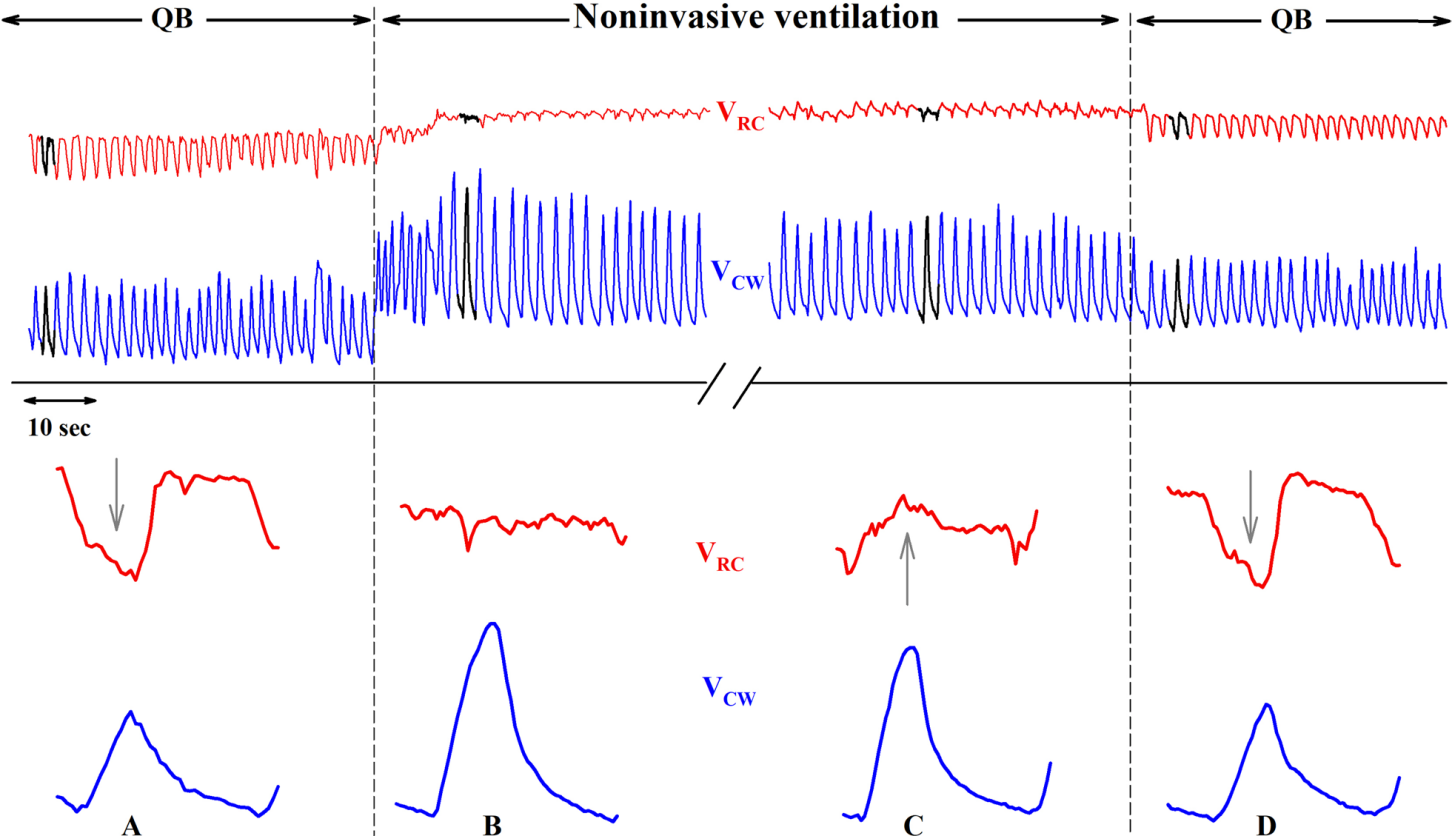
Volume variations of the ribcage (V_RC_) and total chest wall (V_CW_) in an OI type III patient during one minute of spontaneous quite breathing (QB) followed by five minutes of noninvasive ventilation and again one minute of spontaneous breathing after switching off the ventilator (top two tracings). The single breaths indicated by thick lines in the top tracings are shown zoomed in the bottom panels. Data recorded in supine position. Note the presence of an inspiratory paradoxical inward motion of RC during QB (**A**), its reduction immediately after the connection to the ventilator (**B**), the in-phase inspiratory expansion of RC after about 5 min of connection to the ventilator (**C**) and the reappearance of the paradoxical motion of RC immediately after the switching off the ventilator (**D**)

We can speculate that the paradoxical inward movement of the ribcage together with the rapid and shallow breathing pattern promote pulmonary micro-atelectasis in OSA patients that could benefit by nNIV. nNIV is able to overcome upper airways obstruction during sleep, to reverse thoraco-abdominal asynchrony (as shown in Fig. 8) and therefore to recruit the lungs. In this way, nNIV prevents nocturnal hypoventilation and lung atelectasis. However, these are short-lived effects as nNIV is effective only during use, as indicated by the reappearance of the paradoxical motion immediately after the switching off of the ventilator in Fig. 8.

Because our patients showed low dietary quality, in terms of reduced adherence to the Mediterranean diet with low consumption of wholesome foods and higher consumption of noxious foods and confectionery, we believe, and therefore hypothesise, that an adequate dietary intake may break or at least ameliorate the vicious circle. The diet of these patients should include nutrients related to bone health, such as vegetables proteins, vitamins B, D and E, omega-3 fatty acids, oleic acid, selenium, calcium and polyphenolic compounds. The improvement of body composition (i.e. reduction of fat body mass with concomitant increase of lean body mass), as expected by a Mediterranean dietary pattern [48], may have long-lasting ameliorative effects on the quality of life in OI. These comprise: (1) decreasing body weight, truncal and abdominal fat; (2) supplying the adequate energy and micronutrients involved in bone metabolism; (3) reducing the incidence of nocturnal obstructive apnoea [49]; (4) reducing the burden on the thorax while breathing; (5) potentiating pharmacologic therapy (vitamin D and calcium supplement and/or bisphosphonate therapy) and (6) improving physical activity and mobility, particularly during wheelchair transfer.

It is important to reverse this trend, since a high frequency of apnoea during sleep may interfere with restorative sleep that it is known to contribute to negative consequences to health and quality of life. In addition, because of the recurrent fractures, severe OI patients are used to experience pain and fatigue since childhood and therefore they may develop higher level of tolerance, as indicated by the paradoxically good scores of the quality of life questionnaire. Indeed, these answers counteracted the expected low quality of life and higher fatigue [50, 51], but they are also in line with the cross-sectional questionnaire study by Arponen and colleagues [41]. Such difference between physical severity (assessed by physical function) and subjective severity perception (assessed by general health perception) in OIers was recently pointed out by another author [52]. OI patients, therefore, may perceive their physical disorders and limitations differently from health care professionals. We can speculate the higher pain and fatigue thresholds to be one possible cause of OSA to be underestimated in these patients. For this reason, we strongly recommend nocturnal oxygen saturation to be systematically tested, particularly in patients affected by the severe form of the disease who are overweight/obese, with a particularly important neck circumference and who are expected to paradoxically breathing in supine positon.

To our knowledge, this is the first study investigating the nutritional status, body composition and dietary habits in adult OIers. It is also the only one to simultaneously consider three vital functions (nutrition, breathing and sleeping) that have an important impact on the quality of life. Indeed, other authors have separately investigated sleep [12, 13, 41, 44] or respiratory impairment [6–11] or nutrition [17–19, 46, 47]. The latter was shown to negatively impact the psychosocial wellbeing in the OI population and to limit daily physical activity [53]. Our results put in evidence that OI is not only a bone-related disease, but it has also important implications on other disciplines due to its intrinsic characteristics. These results suggest that experts in breathing, in sleep and in nutrition must be part of the multidisciplinary team that should follow an OI patient, particularly in the most severe form [54, 55].

The study has also some limitations. The orthopaedic implants were manually and carefully removed during the analysis of DXA images analysed with the manufacturer software. The increased use of these medical devices in OIers, however, could lead to an incorrect estimation of the body composition results. We used a self-reported questionnaire on the Mediterranean diet as the first simplest way to analyse the dietary pattern of these patients. Of course, the questionnaire may under- or overestimate the perception of the subject. It does concentrate only on some selected dietary aspects without considering the cumulative and interactive effects among dietary components, which reflect the complexity of the human diet. However, this is the reference questionnaire for the Mediterranean diet and it investigates the frequency of consumption of both the protective and less protective foods in many obesity-correlated pathologies [56]. This questionnaire was validated on other pathologies and it was proved to be less time-consuming and cheaper while requiring low patients’ collaboration compared to other more complex methods [57].

The relative low number of patients might be another shortcoming of the study. However, OI is a rare disease and, even if the exact incidence of types III and IV OI is not known, the incidence is much less common than all OI types. In addition, the systematic paradoxical breathing movement and the OSA incidence found were in line with those published on other groups of OIers [8, 12, 13, 41], therefore reinforcing the robustness and the clinical relevance of our results. The physical function was not tested, but it was recently proved to be markedly deteriorated during adulthood, with mobility being significantly different across the types particularly in type III [45]. We have not verified the presence of cranial, cervical and/or mandibular deformities, a recurrent craniofacial alterations in OI, which can predispose toward an anatomically unfavourable upper airway collapse during sleep [58]. Cheung and collaborators have shown that skull base abnormalities is strongly predicted by the clinical severity of OI with an incidence of 22% [59]. The way patients were recruited might be a bias in the selection of the population. Because of their physical condition and limitation, OIers might experience also isolation and negative emotions stemming from a feeling of being different [60]. We can speculate that the patients answering to our social call were more prone to maintain a “fighter” attitude and emotions’ management. This would also explain the good levels of their answers to the quality of life questionnaire. However, when these patients were asked to score their relationship with food, including food choice and motives, dieting, eating relating problems and body perception, they denied answering. Paradoxically, the BES questionnaire was more informative for the non-answering of patients (rather than for the results per se), as this might reveal an altered psychological pathway related to eating patterns to be investigated [61]. Finally, the observational nature of the study offered no proofs about the linkage between breathing, sleeping, and nutrition in OI patients and whether one may lead to the others or vice versa.

Future interventional studies are urged not only to investigate such linkages, but also the potential role of nNIV and of changes of diet in alleviating breathing problems and physical well-being in these patients. These are crucial points that need to be studied remembering that in OI a combination of some intrinsic characteristics of the disease (i.e. thoracic, cranial and mandibular deformities) and some daily habits of the patients (i.e. physical inactivity and low dietary quality) coexists. While the latter can be changed, the former progressively worsen in the most severe forms. It would be also crucial to verify the adherence to nNIV of these patients, as Fig. 8 showed that it had short-lasting effects in reversing paradoxical breathing. If it is not daily used, the effect of nNIV might be therefore misleading.

## Conclusions

This was the first multidisciplinary study that considers breathing, sleeping and nutritional aspects in the most severe forms of Osteogenesis Imperfecta during adulthood. It pointed out that a dangerous vicious cycle ensued among these three functions because of some intrinsic characteristics of the disease and some daily habits of the patients. These bad habits might be partially due to patients’ emotional influence on food choice and self-perception, but also to the lack of guidelines on the physical activity and diet appropriate for OIers. The main consequence is a high incidence of obstructive sleep apnoea, which remains an underdiagnosed disorder in individuals with severe OI who are obese, with a neck to height ratio over than 31.6%, and characterized by paradoxical inspiratory thoracic movement in supine position. The combination of nocturnal non-invasive ventilation associated with controlled dietary regime may break the vicious circle therefore improving the well-being of these patients because “*one cannot breathe well, think well, love well, sleep well, if one has not dined well*” (adapted from Virginia Wolf).

## Data Availability

The data that support the findings of this study are available on request from the corresponding author ALM. The data are not publicly available due to restrictions, since their containing information that could compromise the privacy of research participants.

## Abbreviations

AHI: Apnea–hypopnea index
AUC: Area under the receiver operating characteristic curve
BH: Body height
BMC: Bone mineral content
BMI: Body mass index
BW: Body weight
DEXA: Duel-energy X-ray absorptiometry
FEV_1_: Forced expiratory volume in the first second
FFM: Fat free mass
LMI: Lean mass index
FM: Fat mass
FMI: Fat mass index
FVC: Forced vital capacity
LM: Lean body mass
MED: Mediterranean
nNIV: Nocturnal non-invasive mechanical ventilation
no_OSA: Patients not developing obstructive sleep apnea
ODI: Oxygen desaturation index
OI: Osteogensis Imperfecta
OIQoL: Osteogenesis Imperfecta specific quality of life questionnaire
OI_OSA: Patients developing obstructive sleep apnea
OSA: Obstructive sleep apnea
ROC: Receiver operating characteristic
TLC: Total lung capacity
ΔV_RC_: Ribcage contribution to tidal volume
